# Combining Real-Time Ratings With Qualitative Interviews to Develop a Smoking Cessation Text Messaging Program for Primary Care Patients

**DOI:** 10.2196/11498

**Published:** 2019-03-26

**Authors:** Gina Kruse, Elyse R Park, Naysha N Shahid, Lorien Abroms, Jessica E Haberer, Nancy A Rigotti

**Affiliations:** 1 Division of General Internal Medicine Massachusetts General Hospital Boston, MA United States; 2 Tobacco Research and Treatment Center Massachusetts General Hospital Boston, MA United States; 3 Harvard Medical School Boston, MA United States; 4 Department of Psychiatry Massachusetts General Hospital Boston, MA United States; 5 Department of Prevention and Community Health Milken Institute School of Public Health George Washington University Washington, DC United States; 6 Center for Global Health Massachusetts General Hospital Boston, MA United States

**Keywords:** text messaging, smoking cessation, primary care

## Abstract

**Background:**

Text messaging (short message service, SMS) interventions show promise as a way to help cigarette smokers quit. Few studies have examined the effectiveness of text messaging (SMS) programs targeting smokers associated with primary care or hospital settings.

**Objective:**

This study aimed to develop a text messaging (SMS) program targeting primary care smokers.

**Methods:**

Adult smokers in primary care were recruited from February 2017 to April 2017. We sent patients 10 to 11 draft text messages (SMS) over 2 days and asked them to rate each message in real time. Patients were interviewed daily by telephone to discuss ratings, message preferences, and previous experiences with nicotine replacement therapy (NRT). Content analysis of interviews was directed by a step-wise text messaging (SMS) intervention development process and the Information-Motivation-Behavioral Skills model of medication adherence.

**Results:**

We sent 149 text messages (SMS) to 15 patients. They replied with ratings for 93% (139/149) of the messages: 134 (96%, 134/139) were rated as clear or useful and 5 (4%, 5/139) as unclear or not useful. Patients’ preferences included the addition of graphics, electronic cigarette (e-cigarette) content, and use of first names. Regarding NRT, patients identified informational gaps around safety and effectiveness, preferred positively framed motivational messages, and needed behavioral skills to dose and dispose of NRT.

**Conclusions:**

Patients recommended text message (SMS) personalization, inclusion of e-cigarette information and graphics, and identified barriers to NRT use.

Combining real-time ratings with telephone interviews is a feasible method for incorporating primary care patients’ preferences into a behavioral text messaging (SMS) program.

## Introduction

### Background

There is growing evidence that smoking cessation interventions delivered by mobile phone are effective at helping smokers quit [1]. Smartphone apps have been developed for smokers that deliver evidence-based behavioral advice, acceptance and commitment therapy, mindfulness training, digital photo aging, contextually tailored messages using geoposition and social context, medication adherence support, and positive psychology interventions to name just a few [2-10]. However, so far, no smartphone apps have demonstrated improved long-term cessation outcomes at 6 months or longer.

In contrast, short message service (SMS) text messaging interventions have demonstrated improved long-term cessation among cigarette smokers [1,11-13]. SMS text messaging programs for smokers deliver behavioral advice on the basis of several behavior change theories [14] to increase self-efficacy [15]. Further, they have been shown to improve quit chances by 30% to 70% compared with self-help material or usual care [1,11-13]. Most of the previous mobile health interventions for smokers, both SMS text messaging– and smartphone-delivered interventions, have examined community-based samples recruited from schools or internet advertisements [16-21].

Mobile health interventions for smokers have not been well studied in health care settings. Studies examining mobile apps for smokers in health care settings have not tested long-term outcomes [10,22-24]. Studies of SMS text messaging in health care settings have measured long-term abstinence but have found mixed results. There were 2 studies that examined smoking outcomes among patients and offered varenicline or varenicline plus SMS text messaging and found no effect from adding SMS text messaging [25,26]. Another study found no effect of SMS text messaging for hospitalized smokers [27]. Furthermore, 1 study found no significant effect of SMS text messaging for pregnant smokers [28]. In contrast, 2 primary care–based studies and 1 study among cardiac rehabilitation patients examined SMS text messaging versus usual care or a brief behavioral intervention and found improved smoking outcomes [29,30]. These mixed results highlight the need to better understand how to integrate SMS text messaging in health care settings with other smoking cessation treatments.

All these previous studies targeted motivated or treatment-seeking smokers, yet 80% to 90% of smokers did not meet these criteria [31]. Interventions that actively seek smokers could have a much wider population impact [32]. We have previously examined the feasibility of proactively offering an SMS text messaging intervention to smokers identified from the electronic health record (EHR) of 2 primary care practices [33]. In that study, 10% of the patients including both motivated and unmotivated smokers accepted an SMS text messaging intervention tailored to readiness to quit from their health care system.

Primary care is an important site for delivering tobacco cessation interventions with 84% of US smokers being screened for tobacco use by a physician each year [34]. Receiving digital messages from a trusted source, such as a local health care system [35], may boost their behavioral impact. We also do not know how patients’ expectations for communications from their health care provider affects their preferences for SMS text message content or what literacy level is appropriate for SMS text messages targeting patients.

Integrating SMS text messaging programs within primary care also presents an opportunity to support other treatments including pharmacotherapy. Adherence to smoking cessation medications is suboptimal with nicotine replacement therapy (NRT) users continuing treatment for less than half the recommended duration [36-40]. SMS text messaging programs have been used to improve medication adherence in chronic conditions including HIV, diabetes, and schizophrenia [41-44], but there is only 1 previous study examining an SMS text messaging intervention addressing medication adherence among smokers [25]. In that study, SMS text messages promoting varenicline use among people with HIV did not increase adherence, but abstinence was higher at 8 weeks among patients receiving SMS text messages plus telephone counseling compared with standard care [25].

There are few published studies describing the development and adaptation of smoking cessation mobile health interventions for health care settings [4,22,45-47]. To our knowledge, none have included both behavioral advice and content encouraging NRT adherence for primary care patients. In this paper, we present a step-wise process for message development. Our process followed other published processes for SMS text message intervention design with the unique aspect of combining real-time ratings of messages with daily qualitative interviews with target users [48-50]. This use of real-time ratings is similar to previous work combining behavioral smoking data from ecological momentary assessments with qualitative data to understand substance use behaviors [51].

### Objectives

We aimed to gather insights into primary care patients’ reactions to messages in the context of their daily lives and to understand their experiences with and barriers to using NRT. Specifically, we examined 3 SMS text messaging intervention components: (1) new content for smokers not ready to quit, comprising motivational advice and encouragement to practice quitting, (2) new content promoting NRT use, and (3) content included in an existing national SMS text messaging campaign. The national campaign content is SmokefreeTXT. This content was developed for the US public [14] and was not targeted to patients in primary care settings who may have different expectations for content coming from their health care provider and access to different resources in the primary care context. Our objective was to develop an SMS text messaging program tailored to the needs of smokers in primary care by adapting established SMS text message content and developing new theory-based medication messages, incorporating patients’ preferences for communication from their health care provider, and preferences for language around smoking cessation. Patient interviews and SMS text message assessments were designed to improve our understanding of patient preferences for SMS text messaging and experiences using NRT. These results inform the adaptation of SMS text messages offering behavioral advice to smokers during a quit attempt and the development of novel motivational and medication-focused messages targeting smokers in primary care.

## Methods

**Figure 1:**
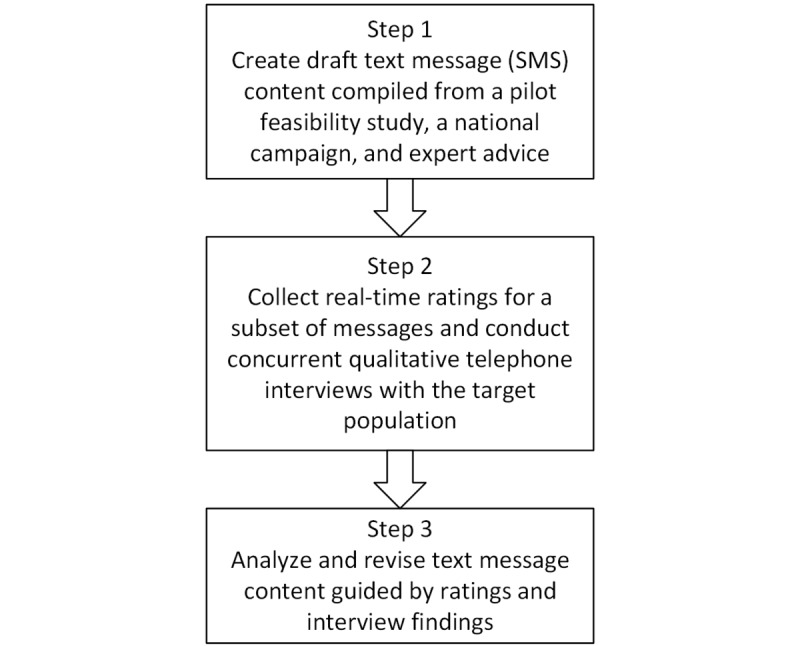
Steps of short message service (SMS) text message development and testing.

Our overall step-wise approach to SMS text messaging intervention development for primary care patients who smoke is shown in Figure 1.

In Step 1, we compiled a preliminary set of programmatic messages for primary care patients who smoke from established sources [33,52]. In Step 2, we asked a sample of primary care patients to rate messages in real time. We measured the time to respond to the rating message to understand when patients were reading and responding to messages. The ratings also measured usefulness or clarity of draft content. We also measured URL links clicked as proportion of URL links clicked out of all URL links sent to a patient to understand engagement with the program and accessibility of Web-based content. The patients simultaneously participated in daily qualitative telephone interviews to explain their ratings, their use of Web-based content, and their preferences for smoking cessation SMS text messaging content. In Step 3, the findings were used to design a set of modifications to the preliminary message set.

### Participants

From February 2017 through April 2017, we recruited smokers from 2 Boston-area community health centers affiliated with a large academic medical center. We recruited patients who participated in a previous feasibility study of SMS text messaging for smokers in primary care [33]. These patients were approved by their primary care providers to be contacted about SMS text messaging research studies for smokers so that we were not required to seek additional provider approval before contacting them. Eligibility criteria included the following: aged 18 years or older, current or former smoker, able to speak and read English, visited their primary care physician in the last 2 years, had a mobile number in their electronic health record, not pregnant, and able to provide informed consent.

### Ethics

The project was approved by the Partners Healthcare Institutional Review Board. Participants provided verbal informed consent to participate and received a US $40 gift card.

### Preliminary Text Messaging Set

A preliminary set of “programmatic messages” comprised messages from 3 sources: (1) the National Cancer Institute’s SmokefreeTXT [52], (2) the novel content we developed for smokers not ready to quit [33], and (3) the novel messages promoting use of NRT based on the Information- Motivation-Behavioral Skills (IMB) model of adherence [53].

#### SmokefreeTXT

Messages from a 2013 version of SmokefreeTXT were used [52]. SmokefreeTXT targets smokers who are ready to quit in the next 30 days. The program invites users to enter a quit date in the next 30 days and sends messages to support them through the quit attempt by addressing motivation, self-regulatory capacity, and other behavioral skills [14]. It includes periodic assessments that query smoking status and other self-reported outcomes and offers real-time support through keywords, which the users can type and send to request specific help with cravings, mood symptoms, or if they *slip* and have a cigarette.

#### Content for Smokers Not Ready to Quit

The content for smokers not ready to quit included motivational and quit induction messages developed for our previous pilot study that aimed to test the feasibility of sending proactive SMS text messages to smokers in primary care [33]. Motivational messages encouraged users to identify personal reasons for change and internal motivations to quit [54,55]. Quit induction messages are used on smokefree.gov and have been studied in randomized trials [52,56]. These messages encourage smokers to try a practice quit attempt (PQA) explained as an attempt to not smoke for hours or days without commitment to increase motivation and self-efficacy [57].

#### Smoking Cessation Medication Adherence Content

Medication-promoting messages were based on the IMB model of medication adherence [53]. This novel content was not included in the previous feasibility study. In the IMB model, information relevant to medication adherence may be accurate or inaccurate and facilitate or hinder adherence and may include how to take medications, medication effectiveness, drug interactions, or side effects. Motivation to adhere to medications encompasses both personal and social motivations and may include the individual’s attitudes toward adherence, beliefs about the effects of adherence, perceived social support to adhere to medication, and interest in complying with the wishes of others. Behavioral skills include the self-efficacy and actual abilities to take medications including acquiring and using medication, dealing with adverse effects, communicating with health care providers, and calling up social support. Preliminary medication-promoting messages included informational messages about the mechanism of action and effectiveness of NRT, motivational reminders highlighting social factors, and behavioral tips about how to use NRT *ad lib* or after a slip.

### Phase 1: Real-Time Message Ratings

From our programmatic message set, we purposively selected subsets of messages with potential challenges for users. First, we selected messages with a high literacy level based on a Flesch-Kincaid score greater than eighth-grade level. Second, we selected messages with URLs. Third, we selected messages describing the PQA and novel medication adherence messages. Using an internet-based mobile messaging platform (Upland Mobile Messaging, Austin, TX), we created 4 sets of 10 to 11 messages scheduled for delivery over a 2-day span between the hours of 9:00 am and 5:00 pm. Each programmatic message was followed by a *rating message* that asked the participant to rate the message’s usefulness or clarity depending on the message content. Each participant was assigned to receive 1 of the 4 sets of programmatic messages and ratings. Assignment to message subsets was sequential, with each message set being rated by 3 to 4 participants.

#### Quantitative Analysis

We compared the characteristics of the participants in this study with those who were unreachable or declined participation using Chi-square and student’s *t* tests. We calculated the proportion of messages rated as clear or useful, the proportion of URL links clicked, and the median and distribution of response times to ratings. Analyses were conducted in Stata version 13 (StataCorp).

### Phase 2: Semistructured Interviews

Each day of messages was accompanied by a qualitative telephone interview. Interviews were conducted by a clinical research coordinator (NS) and a physician-researcher (GK) with qualitative interview experience. Interview topics included structured data on participants’ smoking status, readiness to quit, and use of NRT; they also included open-ended inductive inquiries exploring the day’s real-time message ratings and message content, *a priori* inquiries about preferences for message timing and frequency, personalization, privacy concerns, previous experiences with cessation medications, and *a priori* inquires asking about preferences among sample message types (eg, preference for informational or motivational medication messages, spiritual content, inspirational stories, or games). Interviews were audio recorded and transcribed for content analysis. After every 3 to 4 patients, we iteratively reviewed the transcripts to assess for new content. We stopped recruitment when saturation was reached, defined as the point at which we heard no more new topics or ideas in response to interview questions [58].

#### Qualitative Analysis

Qualitative interview transcripts were content analyzed using NVivo version 11 (QSR International) by 2 coders (GK and NS). The unit of analysis was the patient. Coders first read the transcripts and identified the key concepts. These key concepts were used to develop a preliminary coding framework. Furthermore, the coders reviewed each transcript using the preliminary framework to refine *a priori* themes and add emergent themes [59]. Coding was at the sentence level. All content was analyzed and could be coded with multiple themes. After iteratively analyzing all transcripts and reconciling discrepancies, the final coding structure was reviewed with a third researcher (EP). All interviews were double coded with the final coding structure that included 4 domains, 17 major themes, and 4 subthemes. We used kappa statistics to measure intercoder agreement with the final coding structure. The overall kappa, calculated by averaging across all themes and weighting patients equally, was 80% (individual kappa scores are indicated in Multimedia Appendix 1).

### Phase 3: Modifications to Text Messaging Intervention

In the final phase of this message development process, the qualitative interview findings and ratings informed changes to the SMS text messaging program. To define the message modifications, the study team reviewed the final qualitative themes and, through in-person and written discussion, came to a consensus on the planned message changes.

## Results

### Study Sample

Of 76 participants in the previous feasibility study, 57 (75%, 57/76) were reached and 15 (20%, 15/76) enrolled in this study. Characteristics of the 15 participants are shown in Table 1. Compared with patients who did not participate, participants who enrolled in this study were more often non-Hispanic white (*P*=.04). In all, 9 participants (60%, 9/15) were daily smokers, 4 (26.7%, 4/15) less-than-daily smokers, and 2 (13.3%, 2/15) former smokers who quit after the previous pilot study. A total of 10 (66.7%, 10/15) reported using NRT in a previous quit attempt.

### Phase 1: Real-Time Message Ratings

We sent 149 programmatic messages and 149 rating messages. Of the 24 unique messages with URL links, none were clicked. Participants replied with ratings for 93.2% (139/149) of messages sent. The median time from rating message to reply was 7.0 min (interquartile range 1.0-29.0; Multimedia Appendix 2). Each message was rated by 3.6 participants on average with 1 message receiving only 2 ratings. The 10 missing ratings came from 5 participants. Of the 139 ratings, 96.4% (134/139) rated messages as useful or clear. Messages rated as unclear or not useful included 2 messages describing PQAs, 1 informational message about NRT, 1 motivational message, and 1 high literacy-level message (Multimedia Appendix 3).

### Phase 2: Semistructured Interviews

All 15 participants completed the first qualitative interview, 87% (13/15) completed the second and 2 people were unreachable for the interview despite completing the message ratings. Our interviews produced 17 themes and 4 subthemes across 4 domains (Multimedia Appendix 1).

#### Program Framework

##### Message Frequency and Timing

Participants recommended from 1 to 5 messages per day and some recommended sending messages before bed, in the evening. When asked whether sample messages would be more effective if sent at other times, all participants thought the message’s effectiveness would not be altered if received at a different time. When asked separately about URL links, some participants reported being at work as a reason for not clicking at the time of receipt.

##### Personalization

Most participants liked personalization with their first name and described it as humanizing and comforting. However, several participants had concerns about other types of personalization such as including their doctor’s name:

[Use of first names] makes it sound like it’s not coming from a robot caller.

Daily smoker, female

Yeah, I think [Using your doctor’s name] would feel invasive like, “Whoa, they-- what else do they know about me?”

Former smoker, male

**Table 1:** Characteristics of participants.

Demographic characteristics		Participants (N=15)	Declined or unreachable (N=61)	*P* value^a^
Age (years), mean (range)		46 (28-61)	52 (23-70)	.10
Female, n (%)		6 (40)	41 (67)	.08
**Race and ethnicity, n (%)**				**.04**
	White	12 (80)	58 (95)
	African American	1 (7)	2 (3)
	Latino	2 (13)	0 (0)
	Other	0 (0)	1 (2)
Medical comorbidities^b^		5 (33)	19 (31)	>.99
**Insurance status, n (%)**				**.51**
	Medicare	4 (27)	9 (15)
	Medicaid	4 (27)	12 (20)
	Commercial payer	7 (47)	39 (64)
	Self-pay	0 (0)	1 (2)

^a^On the basis of student *t* test or Fisher exact test.

^b^Includes diabetes, hypertension, and coronary artery disease.

##### Privacy Concerns

Participants reported no issues with privacy of the messages they received. They also reported no concerns for privacy with an SMS text messaging program about smoking and no concerns about other people seeing their messages about smoking.

#### Message Content

##### Electronic Cigarette Content

Participants expected electronic cigarette (e-cigarette) content in messages about other tobacco products or treatments:

The one thing it doesn't include that they may want to include is the electronic cigarettes. Because that's what I used to help me quit and I quit for almost six months...

Daily smoker, female

##### Features-Graphic Content

A few participants recommended adding emoji-style images to attract interest:

Suppose so if you have no time and you look at it and you see a picture, you’ll be more apt to look at it ... make it look fun, have some balloons or something.

Nondaily smoker, female

Moreover, 2 participants recommended adding graphic images of lungs to enhance message effectiveness:

Nobody shows pictures of lungs…They don’t show family members sitting next to the people in bed...I think the shock value of things would really help with people too.

Former smoker, male

##### Specific Facts Versus General Statements About Quitting Tobacco

Participants reported that they found specific statements of the effects of quitting to be more impactful than more general statements:

If there are more specifics on what they’re going to gain out of it and then more specifics on what they’re going to expect doing it, people more likely want to take those steps, knowing what could happen to them.

Daily smoker, female

##### Encouragement and Message Framing

Participants reported that messages offering encouragement and praise would be more effective than negatively-framed messages:

Every couple days you could say, “Well if you didn't smoke, know you can pat yourself on the back.” And just kind of encourage the person and give them good feedback as to, “Good job if you didn't smoke today.” You know give yourself a high-five. As opposed to like, “Don't smoke, this will happen,” and “Don't do that.”

Nondaily smoker, female

##### Language Clarity

Participants did not understand some terminology in the messages including slip, lozenge, trigger, and the PQA:

*Oh, those [lozenges] are the hard candy things?* [Nondaily smoker, male].

##### Language Counseling Versus Coaching

Participants reported counseling for tobacco use had a negative connotation and made it seem more like an illness. Participants were interested in coaching:

I think coaches and-things like are better off because people think of counseling and they think like, “I have mental issue. Oh, I have a drug problem,” or-- “people don't think of cigarettes as heroin or opiates or something like that.”

Former smoker, male

##### URL Links

Participants reported not clicking URL links as they did not have time, were not looking for or needing the information offered, were at work, had no internet access, or lacked computer skills. Participants recommended use of a visual link rather than a URL to increase the appeal. Participants also made suggestions for how to improve messages with URL links such as offering a telephone number for local smoking-cessation programs to learn about available treatment and services in addition to a link to the local program website for those without internet access:

Maybe you could leave a phone number too, something like that...because like I said, I don't have all them fancy phones that can go on the computer.

Nondaily smoker, male

##### Features Games for Distraction

Participants were asked *a priori* questions about preferences for message content from a list of options. Nearly all participants preferred games for distraction:

Progressive things where today you do this, and then tomorrow, you're going to add to your score for this. And then it leads up to you get a silver cup, and then next week, you go for a gold cup...You know how games grab you and bring you in.

Former smoker, male

#### Barriers to Nicotine Replacement Therapy Use

##### Cost

Participants identified several barriers to starting or continuing NRT use including cost, side effects and safety, effectiveness, forgetting, and difficulties or dislikes. Cost was a commonly-cited barrier to using NRT and something participants wanted to receive information about, by SMS text message:

I don't know if they give them free in places. So maybe more information on how you can get them if you don't have money. Because they are pretty pricey.

Nondaily smoker, female

##### Side Effects and Safety

Concerns about NRT side effects and safety included cancer-risk beliefs, risks of smoking when using NRT, and potential for addiction to NRT. These concerns were a source of stress:

I was just like, “Oh my God. If I do smoke with this on, I'm going to like, blow up or something.” So, I just felt like there was a lot of pressure. So, I wanted to smoke more.

Nondaily smoker, female

All these things to help you quit smoking, it's still nicotine going into your body. Can't that still cause you to get cancer?

Nondaily smoker, female

##### Perceived Effectiveness

Some participants reported NRT was ineffective in their previous attempts, and this was a barrier to subsequent use:

[The patches] they're not really great for-- if you smoke a lot and you've been smoking a long time, the patches don't help all that much.

Daily smoker, male

##### Difficulties and Dislikes

Participants described disliking the taste of lozenges and the difficult process of patch disposal:

I mean it's not a real pain in the neck, but they talk about it [the patch] like you got to get rid of it like it's a contaminant. Like it's medical waste or something.

Nondaily smoker, male

##### Forgetting

Few participants reported forgetting medications and some reported feeling aware of having the patch on:

I'm pretty much like “oh my gosh it's on me.”

Nondaily smoker, female

#### Facilitators of Nicotine Replacement Therapy Use

##### Information

Queries about facilitators of medication use were organized around information, motivation, and behavioral skills constructs. People identified informational needs about side effects, safety, and dosing of medications and recommended providing this in simple, short formats:

Maybe, I don't understand how they say if you smoke less than ten cigarettes a day, start on a number two patch. If they explain that a little more.

Nondaily smoker, male

##### Motivation

When asked *a priori* questions about their preferences for informational, motivational, or behavioral skills medication messages, participants who preferred motivational messages described them as caring and conversational:

It's more personal, I don't know. More like let's get to it, it just seemed to me more normal.

Daily smoker, female

##### Behavioral Skills

Participants who liked the behavioral skills messages described them as straightforward and useful. Participants identified needed skills to take NRT such as how to manage slips, *ad lib* use, side effects, and getting refills:

In order not to slip up, take a couple of more-- of the lozenge or the patch. You know what I mean?

Nondaily smoker, male

When asked about tips or skills for remembering to take medications, participants thought reminders by SMS text message could be helpful:

Well, probably if I got a reminder on my phone, a text message or something.

Daily smoker, female

### Phase 3: Modifications to Text Messaging Intervention

On the basis of the interview findings, we modified the existing messages including delivery timing, message text, and pictorial content and added new medication-focused messages for a final program of 244 scheduled messages. We adjusted message timing to add evening messages on some days for a total of 3 to 5 messages per day. Sample messages that were modified or developed based on qualitative data are shown in Table 2. In addition to these changes, we tried to leverage the users' relationship with their health care system by referencing local tobacco-cessation resources. Given the preferences for normal or conversational messages, we added a feature to respond with, “You are welcome” whenever someone texts “Thank you.”

**Table 2:** Description of text message modifications and examples by theme.

Theme		Modification	Example
**Program framework and content**			
	Personalization	We personalized 17 messages to the user’s first name. Other types of personalization were not used, such as referencing the user’s primary care provider.	You are getting closer to the big day [first_name]. It may help to cut back on the number of cigarettes you smoke. Give it a try.
	Electronic cigarette (e-cigarette) content	We added messages that acknowledged that people are using electronic nicotine delivery systems.	Using e-cigs or vaping? We don't know if these help people quit cigarettes. Keep your smokefree goal in mind--to quit cigarettes completely.
	Graphic content	We added emoji icons to 7 messages. We added links to personal stories from the Center for Disease Control’s “Tips from former smokers” public health campaign and links to the World Health Organization's library of graphic warning labels.	Wow, 3 weeks smokefree. [balloon emoji] Give yourself a pat on the back! Just don't light up to celebrate; that is a slippery slope.
	Specific facts versus general statements	We added messages with facts about the effects of quitting smoking.	Quitting smoking improves your health immediately, it lowers your blood pressure in the first 20 minutes.
	Encouragement and framing of messages	We used participants’ own language to replace negatively-framed messages with encouraging messages.	Wow, 2 weeks smokefree! Ask the person next to you for a high five! You did well! You deserve it.
	Language clarity	We added definitions of triggers, lozenges, and slips and modified our description of the practice quit attempt.	You might slip by having a puff or even 1 or 2 cigarettes after you quit. Don't let 1 slip be an excuse to start smoking again. Learn from the situation ASAP and move on.
	Language counseling versus coaching	We edited all messages to use the words “coach” or “coaching” instead of “counseling.”	Quit-Tobacco coaches & medication can increase your chances of quitting. Free 1-on-1 coaching is available at MGH Community Health Centers. Call XXX-XXX-XXXX for more info.
	Features: URL links	We modified the link content to reflect requested information such as information about e-cigarettes or patch dosing. We added telephone numbers together with URL links to additional tobacco treatment resources.	Using e-cigs or vaping? We don't yet know if vaping is safe or if it helps to quit smoking. Nicotine patches are safe & effective. Learn more: URL.
	Features: games for distraction	We created a trivia game prompted by a keyword TRIVIA.	Distract yourself with trivia for a few minutes. When did MGH open its doors? Text A for 1801, B for 1821 or C for 1905; <B response> That's right! [gold cup emoji] MGH opened in 1821. It is the 3rd oldest general hospital in the US.
**Medication information, motivation, and behavioral skills**			
	Information: dosing	We added messages with simple dosing instructions.	Patch users, if you smoke 10 or more cigs per day start with step 1. If you smoke less than 10 cigs start with step 2: URL.
	Information: safety and side effects	We added messages with information about the maximum daily dose to reassure participants concerned about overuse and describing the low risk of addiction to nicotine medications. We also added messages with advice for dealing with common side effects of skin irritation or sleep disturbance.	The nicotine patch and lozenge have less nicotine than cigarettes. You are not likely to become addicted to the patch or lozenges.
	Information: medication effectiveness	We added messages encouraging users to consider combination therapy in consultation with their doctor to address concerns of ineffectiveness and advice on correct medication use to maximize effectiveness.	Consider using the patch and gum or lozenge together if you’ve been unable to quit with medication in the past. Ask your doctor for advice.
	Motivation: forgetting	We added weekly reminder messages offering conversational encouragement and asking users if they used medications that day.	We hope you are doing well. Did you use your nicotine patch or lozenge today? Reply with USED or NOT USED.
	Motivation: social support	We added motivational messages highlighting social motivations.	Nicotine patches and lozenges increase your chance of quitting, which will protect your health and help you to be there for your family.
	Behavioral skills: cost	We added messages describing behavioral skills including checking on insurance coverage and contacting the local quit-line which has free medication opportunities.	Your insurance may cover quit smoking medications. To learn more about your options, speak with your doctor & visit: URL.
	Behavioral skills: difficulties and dislikes	We added a message about safe disposal, labeled as a tip instead of a rule or regulation.	Tip: Save the pouch your nicotine patch came in. Fold used patches sticky sides together and throw them out in the pouch safely away from kids & pets.

## Discussion

This study aimed to develop an SMS text messaging program tailored to readiness to quit using preferences of primary care patients who smoke cigarettes and to explore patients’ previous experiences with NRT with the purpose of developing messages promoting NRT use.

### Principal Findings

By combining real-time message ratings with daily interviews, we identified SMS text message modifications including preferences for the inclusion of graphics, expectations around e-cigarette content, preferences for the inclusion of personalization by user’s name, and recommendations to make URL links more impactful by using pictures or adding telephone numbers for those without internet access. Real-time ratings provided feedback, in most cases, within 30 min of receiving the message. We also identified preferences for message style such as a conversational tone and use of emoji graphics. Participants described barriers to taking smoking-cessation medications including costs, side effects and safety concerns, and perceived effectiveness.

### Comparison With Previous Work

Many previous mobile health apps and SMS text messaging interventions used focus groups, interviews with individuals in the target population, or professional input to develop message content [4,46,47,49,50,60]. Our work used a hybrid approach on the basis of recommended steps for SMS text messaging program development [61]. Few previous studies have combined real-time assessments of SMS text messages with daily interviews [60]. Our interviews provided insight about what patients were doing when they received messages and their reaction in that setting. Although we used this real-time rating for intervention development, it has also been used within interventions through machine-learning and 5-item real-time user ratings to select messages that influence smoking cessation behaviors [62].

In this study, when presented with different options for personalizing messages, participants liked personalizing SMS text messages with their first names, but the use of the physician’s name was viewed as intrusive by some. Previous work examining preferences for SMS text messages about health topics has produced conflicting results, with some participants expressing concerns about privacy of message content about health screening tests, whereas others expressed no concern despite the inclusion of sensitive material such as HIV status [63,64]. We tried to balance privacy concerns by using first names but excluding personal information described as intrusive. For example, instead of referencing the individual physician, we included the name of the local health care system [65].

Previous work has explored the effectiveness of graphic images or emoticons in nutrition campaigns [66,67]. Previous work has also shown that individuals communicate about tobacco products via social media using emoticons or images [68]. To our knowledge, this is the first study describing user preferences for graphic images or emoticons in an SMS text messaging program for smokers. It is possible that proactively sending SMS text messages with a link to graphic images may confer some of the benefits graphic warning labels confer on smoking cessation [69].

### Limitations

Our sample recruited participants from an earlier SMS text messaging feasibility study. All of them had previously seen a smoking cessation SMS text messaging program; this experience may have introduced bias. Several were former smokers. We tested only a subset of messages over 2 days and did not gather participants’ reactions to the entire SMS text messaging program. We used a 2-item rating scale for simplicity and with this scale, most of the messages were rated positively. Use of a nonbinary rating instrument, changing the rating system to reflect the targeted behavioral constructs such as self-efficacy, or rating usability [50] may produce greater insight into message preferences and impact.

### Conclusions

This message development method of combining message ratings with daily telephone interviews is novel and was feasible among a sample of smokers in primary care. This method produced insights and modifications to the SMS text messaging intervention, including edits to the message style such as addition of graphics, conversational tone, editing of URL links, and clarifying the language. User-reported barriers and facilitators of NRT use were used to generate informational messages about medication safety, use and effectiveness, motivational messages in a conversational style, and messages describing behavioral skills such as dealing with slips when on NRT.

Combining real-time SMS text message ratings with qualitative data was feasible among primary care patients who smoke, directed modifications to SMS text message content to better tailor it to primary care patient preferences, and was used to produce novel medication-adherence messages. The final SMS text messaging program is being tested in a pilot randomized trial of SMS text messaging and mailed NRT among primary care patients who smoke (NCT03174158).

## Appendix

Multimedia Appendix 1 Qualitative themes and kappa statistics.

Multimedia Appendix 2 Distribution of response time to message rating queries.

Multimedia Appendix 3 Rating message responses (N=149).

## Abbreviations

e-cigarette: electronic cigarette
EHR: electronic health record
IMB: Information-Motivation-Behavioral Skills
NRT: nicotine replacement therapy
PQA: practice quit attempt
SMS: short message service
