# Supplementary material for: Combining Real-Time Ratings With Qualitative Interviews to Develop a Smoking Cessation Text Messaging Program for Primary Care Patients
Source: JMIR Mhealth Uhealth. 2019 Mar 26;7(3):e11498. doi: 10.2196/11498 (PMC6454345; doi:10.2196/11498)
Supplement: Multimedia Appendix 1 [file mhealth_v7i3e11498_app1.docx]

| Domain | Theme | Kappa statistic^a^  % |
| --- | --- | --- |
| Program Framework | Message frequency and timing | 91.5 |
|  | Personalization | 91.6 |
|  | Privacy concerns | 77.7 |
| Message content | Electronic cigarette information | 74.3 |
|  | Desired features | 77.5 |
|  | Specific facts versus general statements | 88.5 |
|  | Encouragement and framing of messages | 79.7 |
|  | Language | 74.5 |
|  | URL links | 93.6 |
| Barriers to NRT use | Cost | 89.8 |
|  | Side effects and safety | 82.4 |
|  | Perceived effectiveness | 71.7 |
|  | Difficulties and dislikes | 75.1 |
|  | Forgetting | 80.5 |
| Facilitators of NRT use | Information | 93.5 |
|  | Motivation | 73.0 |
|  | Behavioral skills | 84.8 |

eTable 1. Qualitative themes and kappa statistics

^a^ Kappa averaged across sources with sources weighted equally.
