# Supplementary figures and images for: Combining Real-Time Ratings With Qualitative Interviews to Develop a Smoking Cessation Text Messaging Program for Primary Care Patients
Source: JMIR Mhealth Uhealth. 2019 Mar 26;7(3):e11498. doi: 10.2196/11498 (PMC6454345; doi:10.2196/11498)

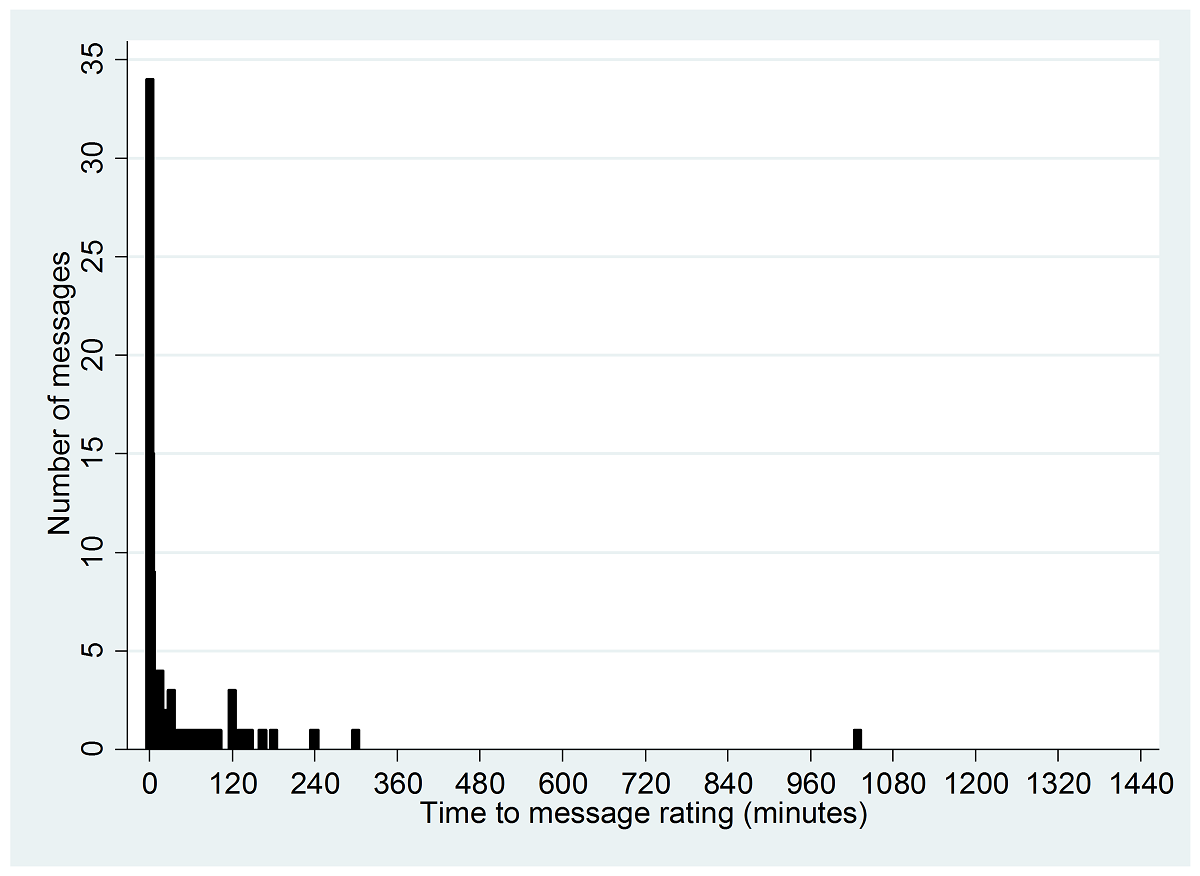

Supplement: Multimedia Appendix 2 [file mhealth_v7i3e11498_app2.png]
