# Supplementary material for: Combining Real-Time Ratings With Qualitative Interviews to Develop a Smoking Cessation Text Messaging Program for Primary Care Patients
Source: JMIR Mhealth Uhealth. 2019 Mar 26;7(3):e11498. doi: 10.2196/11498 (PMC6454345; doi:10.2196/11498)
Supplement: Multimedia Appendix 3 [file mhealth_v7i3e11498_app3.docx]

eTable 2. Rating message responses (N=149)

| Rating message response | N | % |
| --- | --- | --- |
| Usefulness ratings | 131 |  |
| Useful |  | 91.6 |
| Not useful |  | 0.8 |
| Missing |  | 7.6 |
| Clarity ratings | 18 |  |
| Understood completely |  | 77.8 |
| Not understood completely |  | 22.2 |
| Missing |  | 0.0 |
